# Sedation and Analgesia in Children with Developmental Disabilities and Neurologic Disorders

**DOI:** 10.1155/2010/189142

**Published:** 2010-07-20

**Authors:** Todd J. Kilbaugh, Stuart H. Friess, Ramesh Raghupathi, Jimmy W. Huh

**Affiliations:** ^1^Department of Anesthesiology and Critical Care, Children's Hospital of Philadelphia, University of Pennsylvania School of Medicine, Philadelphia, PA 19104, USA; ^2^Department of Neurobiology and Anatomy, Drexel University College of Medicine, 2900 Queen Lane, Philadelphia, PA 19129, USA

## Abstract

Sedation and analgesia performed by the pediatrician and pediatric subspecialists are becoming increasingly common for diagnostic and therapeutic purposes in children with developmental disabilities and neurologic disorders (autism, epilepsy, stroke, obstructive hydrocephalus, traumatic brain injury, intracranial hemorrhage, and hypoxic-ischemic encephalopathy). The overall objectives of this paper are (1) to provide an overview on recent studies that highlight the *increased* risk for respiratory complications following sedation and analgesia in children with developmental disabilities and neurologic disorders, (2) to provide a better understanding of sedatives and analgesic medications which are commonly used in children with developmental disabilities and neurologic disorders on the *central nervous system.*

## 1. Introduction

With advances in health care, many children with developmental disabilities and neurologic disorders are living longer lives, and increasingly require diagnostic and therapeutic interventions. Pediatricians and pediatric subspecialists are increasing being called upon to safely sedate and provide analgesia for these children for diagnostic procedures (CT, MRI, angiogram, endoscopy, and bronchoscopy) and for therapeutic interventions (interventional radiology, intracranial injury, and emergency stabilization). This paper will focus on children with developmental disabilities and neurologic injury, and will highlight the risks involved with these patients, and the effects of common sedatives and analgesic agents on the central nervous system. The purpose of this paper is to provide the pediatrician and pediatric subspecialist a better understanding on the neurologic effects of different sedative and analgesic medications so that rational and safe choices can be used in children with developmental disabilities and neurologic disorders without causing further “neurologic” compromise.

## 2. Materials and Methods

We performed an extensive review of the medical literature regarding sedation analgesia in children with developmental disability and neurologic disorders utilizing Pubmed. Search terms included “sedation”, and “analgesia”, “pediatric”, “child”, “neonate”, “brain”, “developmental disabilities”, “neurologic”, “autism”, “epilepsy”, “seizure”, “stroke”, “hydrocephalus”, “traumatic brain injury”, “intracranial hemorrhage”, “hypoxia-ischemia”, and “encephalopathy” and the period of search was from 1960–2010. The authors are pediatric neurocritical care specialists and have extensive clinical experience caring for pediatric patients with developmental disabilities and neurologic disorders and research experience in experimental animal models of pediatric neurologic injury.

## 3. Results and Discussion

### 3.1. Overview—Increased Risk for Respiratory Complications following Sedation in Children with Developmental Disabilities and Neurologic Disorders

Sedation and analgesia for the pediatric patient with developmental disabilities and neurologic disorder require a thorough understanding of potential adverse events, and the knowledge and skill to avoid potentially life-threatening complications from the administration of sedative and analgesic medications. In addition, the practitioner must focus particular attention on the entire periprocedural period including presedation evaluation, sedation/analgesia administration, and recovery. The American Academy of Pediatrics, Section on Anesthesiology has published *Guidelines for the Pediatric Perioperative Anesthesia Environment,* which includes suggestions for age categorization, need for intensive care following sedation for recovery, and presence of coexisting disease [1]. 

Since these guidelines were published, sedation outside of the operating room continues to increase, along with the varied practitioner's disciplines that are delivering sedation. With this practice increasing, the debate about safety and the practitioner core competency requirements to provide sedation and/or analgesia to the complex pediatric patient with developmental disabilities and neurologic disorders has also increased and several policy statements have been published by different professional societies [2], with no clear evidence of practice standards and incidence of adverse outcomes. To aid in the investigation of the practice and potential adverse outcomes associated with the delivery of sedation outside the operating room, the Pediatric Sedation Research Consortium (PSRC), a collection of 37 institutions that share information on sedation practices within their individual institutions, has created a self-reporting prospective, observational database. This database has provided vital information to define the frequency and nature of adverse events during pediatric sedation from a multispecialty perspective [3]. Large PSRC studies have shown a relatively low risk to pediatric sedation by practitioners other than anesthesiologists [4]. However, despite zero deaths in the 49,836 sedation encounters, one in 65 of these sedation encounters was associated with stridor, wheezing, airway obstruction, laryngospasm, or central apnea, conditions that all have the potential to deteriorate to respiratory failure and death. Airway obstruction and pulmonary complications were the most frequently cited adverse event. In subsequent analysis, factors that related to higher rates of pulmonary complications were young patients, use of adjunctive opiates, and patients with a higher American Society of Anesthesiology (ASA) status (≥III), a large proportion with *neurologic conditions* [5]. This continues to emphasize that pediatric patients with neurologic disorder and developmental disabilities receiving sedation continue to be at increased risk for adverse events with the most prevalent concerns for airway obstruction and altered respiratory mechanics. Unfortunately, extensive studies have not been performed to identify specific patients at risk and aid in the development of evidence-based clinical protocols for patients with neurologic pathology and developmental disabilities. Most reported experience refers to scattered case reports of specific syndromes (Butler et al. has an excellent review of sedation complications related to many specific syndromes [6]).

So what are the actual added risks associated with sedation of the pediatric patient with developmental disabilities or neurologic disorders? Brain MRI has become an important diagnostic and management tool for these children and is being increasingly used in many pediatric centers [7]. Kannikeswaran and colleagues recently published a retrospective review of children, 1–18 years of age, sedated for brain MRI with and without developmental disability [8]. Developmental disability is defined by these investigators as delay in one of the following: fine/gross motor, cognitive, speech/language, social/personal, and activities of daily living. Pentobarbital and fentanyl were the two most common medications used with no difference in mean dosages between children classified as “normal” or “developmental disability”. However, the patients classified as having developmental disability had a threefold increased incidence of hypoxia (11.9% versus 4.9%; *P* < .01). These findings seem to recapitulate the findings described in the PSRC studies: an increase in adverse events, most notably airway compromise, for children with developmental disabilities and those with neurologic disorders. In this study, the most common diagnosis for the cause of developmental disability was autism (36%). In addition, the authors included patients with attention deficit hyperactivity disorder (20%) as a diagnosis for developmental disability. 

In another study, a small observational chart review performed by Elwood et al. suggests that the anteroposterior oropharyngeal airway diameter was smaller in children with developmental delay than in those without developmental delay, in static MRI images [9]. The limitations in this study were the varied diagnoses within groups of patients diagnosed with developmental delay without specific recommendations for certain patient populations. However, it does reinforce the idea that sedation practitioners need to exhibit marked vigilance for airway patency in patients with developmental disabilities. In addition to a baseline risk for airway compromise in patients with developmental disability, Cortellazzi and colleagues showed that the risk for airway obstruction significantly increased in neurologically impaired children undergoing MRI who were administered a combination of sedative medications [10]. Thus, as practitioners escalate pharmacologic intervention a patient with developmental disabilities or neurologic disorders is at increased risk for airway obstruction and may need higher level of care, including the potential need for a pediatric emergency medicine specialist, anesthesiologist, or intensivist.

The choice of sedation plan varies by institution, practitioner credentials, and experience. Protocols are based on agent pharmacokinetic and pharmacodynamic profiles, with an attempt to maintain a proper plane of sedation and analgesia without respiratory and hemodynamic compromise. However, virtually no protocol exists on the use of different sedatives and analgesic medications with the focus on preventing “neurologic” compromise. While there is not enough evidence-based data to support specific clinical guidelines for sedation and analgesia in children with developmental disabilities and neurologic disorders, the authors' hope is that the sedating practitioner will have a better understanding to safely administer these medications without promoting “further” neuronal injury. 

### 3.2. The Central Nervous System Effects of Different Sedative and Analgesic Medications Commonly Used in Children with Developmental Disabilities and Neurologic Disorders

The practitioner must have a well-developed understanding of the effects that different sedative and analgesic agents will have on cerebral vasculature, metabolism, autoregulation (maintaining constant cerebral blood flow despite changes in perfusion pressure), intracranial pressure (ICP), and cerebral perfusion pressure (the perfusion pressure that causes blood flow to the brain); see Table 1 that provides the route and dosages of the different sedative and analgesic medications that are commonly administered. 

#### 3.2.1. Opioids

Opioids, such as morphine, fentanyl, and remifentanil, have long been considered effective adjuvant medications for analgesia of patients with developmental disabilities and neurologic disorders. Opioids are very useful in the treatment of nociceptive pain, and are crucial in developing a balanced sedation plan when analgesia is a concern, for example, intubation and comorbid injuries. Higher doses of opioids can also have some degree of sedation and even hypnosis; however, sedation is a side effect and not the intended pharmacodynamic purpose. Furthermore, opioids lack amnestic properties, and therefore are rarely used as sole agents for sedation in children. Opioids are commonly coadministered with benzodiazepines, because of their ability to provide sedation, amnesia, and hypnosis. Side effects of all opioids are similar, and include: constipation, urinary retention, sedation, nausea, vomiting, respiratory depression, bradycardia, hypotension, and pruritis. 

Opioid pharmacology can have effects on the central nervous system. Cerebral metabolic oxygen rate, cerebral blood flow (CBF), and ICP all decrease with the administration of opioids if a patient's arterial carbon dioxide (PaCO_2_) remains unchanged. An increase in PaCO_2_ relaxes smooth muscle, dilates cerebral vessels, decreases cerebrovascular resistance, and increases CBF [11]. However, opioids have minimal effects on cerebral hemodynamics in adequately resuscitated patients with controlled ventilation [12]. The use of short-acting and ultra-short acting IV narcotics (fentanyl, sufentanil, and remifentanil) via bolus infusion and/or continuous infusion reported conflicting data on ICP effects [13–15]. Opioids have a direct effect on the respiratory centers in the medulla, and decrease minute ventilation by decreasing respiratory rate and produce a dose-dependent depression of ventilatory response to carbon dioxide levels; therefore, opioids decrease the apneic threshold which may lead to hypoxia and respiratory failure. This side effect may be exacerbated in children with developmental disabilities and neurologic disorders, who commonly have hypotonia, central apnea, and inadequate airway reflexes. 

Morphine, like most narcotics tend to decrease heart rate, depending on the level of sympathetic output, through central vagal stimulation. In addition to a negative chronotropic effect, morphine can lower mean arterial blood pressure via arterial and venous dilation. Venodilation lasts longer than arterial dilation and at increasing dosages will decrease cardiac output and lower myocardial oxygen demands. Because of these properties morphine is commonly administered in adults with myocardial ischemia; however, in patients with traumatic brain injury a substantial decrease in cardiac output and cerebral perfusion pressure may lead to cerebral ischemia. Morphine administration results in elevated histamine levels released from non-IgE-mediated stimulation of mast cells. Histamine can result in decreased systemic vascular resistance and an increased incidence of pruritis in children. In children with developmental disabilities and neurologic disorders it may be difficult to differentiate between increasing levels of agitation due to pain or pruritis, and other side effects. The terminal half-life of morphine is higher in neonates, especially preterm neonates, and decreases with age; however, there is significant individual variability in children. On average the terminal half-life (*t*_1/2_) is approximately 9 hours in preterm infants, 6.5 hours in full-term neonates, and 2 hours in infants and children. 

Fentanyl has several advantages over morphine as an adjuvant medication for analgesia in children with developmental disabilities and neurologic disorders. Fentanyl crosses the blood brain barrier quickly and has a rapid onset and relatively short offset. In lower doses, fentanyl has minimal effects on cardiac output or respiratory depression unless used in combination with other medications such as benzodiazepines. Fentanyl is highly lipophilic and can be administered by intranasal, transmucosal, or transdermal routes. One of the major side effects of fentanyl is that *rapid* IV bolus can cause chest wall rigidity. In our intensive care unit, fentanyl is the most common opioid used to provide analgesia in postoperative patients with developmental disabilities and neurologic disorders and is administered over 3 to 5 minutes when administered by the IV route. We also commonly administer fentanyl in combination with midazolam to provide sedation and analgesia for intubation and mechanical ventilation.

Remifentanil is a potent ultra-short acting synthetic opioid that has become a common component of total intravenous anesthetics (TIVAs) especially for procedures requiring neuromonitoring of somatosensory evoked potentials (SSEP) and motor evoked potentials (MEP) in children for neurosurgery and spinal surgery. The half life is 4 minutes, and unlike other synthetic opioids which are metabolized by hepatic elimination, remifentanil is metabolized by nonspecific tissue and plasma esterases, thereby eliminating accumulation. Remifentanil is also commonly used in combination with other sedatives such as propofol or midazolam for short painful procedures [16]. Significant dose-dependent bradycardia can be associated with remifentanil administration. Due to the lack of effect on neuromonitoring, lack of accumulation, and short half life, remifentanil can be used with great success by allowing the practitioner the ability to quickly adjust the depth of sedation for patients with neurologic disorders [17].

#### 3.2.2. Benzodiazepines

Benzodiazepines are particularly useful for sedation in pediatric patients with developmental disabilities and neurologic disorders because of their pleiotropic effects: sedation, anxiolysis, muscle relaxation, and anterograde amnesia. Benzodiazepines also have anticonvulsant effects by enhancing the effect of the neurotransmitter gamma-aminobutyric acid (GABA) and is ideal for sedating children with epilepsy. Significant effects on organ systems include: decrease in blood pressure, depressed ventilation (transient apnea, especially in combination with opioids), and a decrease in cerebral metabolic rate. It is very interesting to note that basic science research continues to try to elucidate the effect of anesthetics, including benzodiazepines, on the developing brain and neurocognitive function [18]. No traumatic brain injury studies, solely involving pediatric patients, exist on the administration of commonly used benzodiazepines (midazolam, lorazepam, diazepam). One case series studied the effects of diazepam in 7 adults and 1 adolescent with severe traumatic brain injury and revealed a reduction in cerebral blood flow and cerebral metabolic rate with no effect on blood pressure [19]. In our ICU, if the fentanyl fails to control intracranial hypertension, and as long as the hemodynamics are adequate, we will then commonly administer a bolus IV dose of midazolam and start a continuous IV infusion in a critically ill patient who is intubated and mechanically ventilated.

Midazolam is a short-acting (unlike lorazepam and diazepam), water-soluble benzodiazepine commonly used for sedation in children with developmental disabilities and neurologic disorders. Midazolam does not cause local irritation after injection (unlike diazepam) and can be readily mixed with other medications for intravenous, intramuscular, oral, intranasal, or rectal administration. Rapid redistribution from the brain to other tissues, and rapid metabolism by the liver, accounts for a short duration of action and an elimination half life of 1 to 2 hours [20].

#### 3.2.3. Barbiturates

Barbiturates, such as thiopental and pentobarbital, have long been considered effective sedatives for patients with neurologic disorders. Barbiturates decrease cerebral blood flow (CBF) and cerebral metabolic rate (CMRO_2_) in a dose-dependent fashion but preserves autoregulation [21]. Reductions in CBF and CMRO_2_ result in a reduction in ICP which can be useful for traumatic brain injury patients as well as acute stroke or intracranial hemorrhage patients with intracranial hypertension. In animal studies, barbiturates have been observed to reduce infarct size following focal cerebral ischemia [22]. Barbiturates' neuroprotective characteristics are also related to reductions in ischemia-induced glutamate release and inhibition of intracellular calcium release [23, 24]. Barbiturates have anticonvulsant properties and are ideal for sedating children with seizure disorders.

Pentobarbital is a commonly used barbiturate for sedation in children undergoing diagnostic radiologic imaging. It can be administered orally or intravenously. Recovery time can be prolonged following administration which may be a concern if subtle changes in neurologic exam need to be detected such as in the acute pediatric stroke patient. Some pediatric patients may also experience severe agitation during recovery which can confound sequential neurologic examinations [25]. High-dose pentobarbital is also used as a third tiered therapy to control intracranial hypertension following severe traumatic brain injury in children but the practitioner must be aware of the association with hemodynamic instability and the need for blood pressure support with intravenous fluids and inotropic infusions [26].

Besides myocardial depression and hypotension, barbiturates also have the side effect of respiratory depression which can lead to hypoxia. This side effect may be exacerbated in children with developmental disabilities. In a retrospective study, 260 children with developmental disabilities and 226 children without developmental disabilities undergoing brain MRI with sedation were reviewed. No difference in dosages of pentobarbital were observed between the two groups but there was a threefold increased incidence of hypoxia in children with developmental disability (11.9% versus 4.9%) [8]. In summary, pentobarbital is commonly used for sedation in children undergoing diagnostic radiologic procedures such as MRI or CT scan, but in the child with developmental disabilities or neurologic disorders the practitioner must be aware of the important side effects of systemic hypotension and respiratory depression and be prepared to respond to them quickly to avoid further neurologic injury.

Thiopental is an ultrashort-acting barbiturate which in its IV form is commonly used as an induction agent for intubation. The fast onset for induction and short duration of effect make it an attractive agent for rapid sequence intubation in pediatrics. It still poses some of the risks associated with barbiturates including myocardial depression and hypotension. The use of rectal thiopental (15–25 mg/kg) for sedation of pediatric patients undergoing diagnostic CT imaging of the head has been reported to be effective [27]. Due to the short duration of action, thiopental is generally not used for sedation during MRI imaging due to longer scan times.

#### 3.2.4. Etomidate

Etomidate is a short-acting IV drug that produces sedation, anxiolysis, and amnesia. Side effects include respiratory depression, hypotension, myoclonus, and adrenal suppression. Etomidate has the benefits of decreasing ICP by reductions in CBF and CMRO_2_ and has the advantage of producing less cardiovascular depression than barbiturates or propofol, and preserving cerebral perfusion pressure [28, 29]. These neuroprotective qualities are counterbalanced by its ability to increase cerebral vascular resistance by a greater magnitude than its reduction of CMRO_2_ resulting in an increased metabolic deficit [30, 31]. The increased metabolic deficit has the potential to expand the ischemic core and penumbra in brain-injured tissue. This increase in cerebrovascular tone is thought to be attributed to etomidate's inhibition of nitric oxide synthase [32].

Previous reports on the efficacy of etomidate for pediatric sedation during diagnostic imaging have been mixed. Kienstra et al. performed a prospective randomized trial comparing etomidate versus pentobarbital for head and neck CT imaging in children 6 months to 6 years [33]. Sedation success rate was significantly lower in the etomidate group (57%–76% versus 97%) but the duration of sedation was not surprisingly shorter in the etomidate group. Prospective data collected from the PSRC also compared sedation with pentobarbital versus etomidate for diagnostic CT [34]. Only 1 of 446 children sedated with etomidate was deemed “not ideal sedation” compared to 11 of the 396 children who received pentobarbital and duration of sedation was shorter with etomidate (34 versus 144 minutes). Etomidate may have a role for sedation during diagnostic CT imaging but etomidate's short duration of sedation is disadvantageous for the longer scan times required for MRI. Of further note, etomidate's role in the sedation of the pediatric stroke and intracranial hemorrhage patient may be limited and caution should be used to avoid further expansion of the penumbra and ischemic core.

#### 3.2.5. Ketamine

Ketamine is a phenylcyclidine derivative typically formulated as a mixture of two enantiomers in a hydrochloride salt form. It possess low pH of around 4 which can produce pain at the injection site when administered intramuscularly or intravenously. Ketamine can provide both sedation and analgesia. Ketamine is a *N*-methyl-*D*-aspartate (NMDA) antagonist which produces increases in CBF and CMRO_2_ [35, 36]. Early studies in patients with obstructed CSF pathways reported ketamine administration increased ICP with reductions in cerebral perfusion pressure [37, 38]. More recent studies in adult patients with severe head injury have demonstrated improvements in cerebral perfusion pressure and minimal increases in ICP with ketamine [39–41]. One recent report of 30 intubated pediatric head injury patients observed that single doses of ketamine lowered ICP without producing decreases in blood pressure or cerebral perfusion pressure [42]. However, it is still unclear regarding the effect of ketamine on ICP in patients where ventilation is not being tightly controlled. At the present time, there is not enough data to recommend the use of ketamine in the pediatric population at risk for intracranial hypertension, but further studies to assess the role of ketamine in this patient population are warranted. Ketamine is sometimes used as a continuous infusion in intubated patients as an anti-convulsant for children with refractory epilepsy. While ketamine is also a bronchodilator and is helpful in children with asthma, it increases oropharyngeal and airway secretions which may be problematic in children with neurologic disorders who have difficulty handling respiratory secretions. As a result, pretreatment with an antisialogogue such as glycopyrrolate or atropine before the administration of ketamine may be beneficial. In older children and adolescents, hallucinations and delirium can occur; these patients are often premedicated with a short-acting benzodiazepine such as midazolam.

#### 3.2.6. Propofol

Propofol is a short-acting sedative-hypnotic IV agent that can be used to provide moderate or deep sedation. Propofol can induce a deep state of sedation rapidly, provide a short duration of effect, and have a pleasant recovery phase [43]. Propofol is a very popular agent for sedating pediatric patients with neurologic conditions for noninvasive diagnostic imaging, such as a CT scan or MRI. Due to the fast onset and recovery following administration, repeated neurologic examinations are easy to assess such as a child with sickle cell disease who comes in with altered mental status due to a stroke. Propofol also has anticonvulsant properties and reduces ICP which can be advantageous in sedating a patient with epilepsy or a patient with concerns for obstructive hydrocephalus due to a malfunctioning ventriculoperitoneal shunt to obtain diagnostic neuroradiologic imaging [44–46]. While there have been cases of propofol providing adequate sedation and successfully treating intracranial hypertension [47, 48], several pediatric traumatic brain injury case reports have reported metabolic acidosis and death in patients on prolonged (24 hrs) continuous infusion of propofol [49–53]. In the 2003 published guidelines for the care of severe pediatric traumatic brain-injured patients, “continuous infusion of propofol is not recommended” [54]. 

 Adverse effects of propofol include pain at the injection site, apnea or respiratory depression, hypotension, and bradycardia which can be detrimental in a patient at risk for brain ischemia. Propofol does not provide any analgesia. As already discussed, a rare but potentially fatal “propofol infusion syndrome”, associated with lactic acidosis, hyperlipidemia and multiorgan system failure was first described in pediatric patients who received prolonged (24 hours) continuous infusion and at higher dosages (>4.5 mg/kg/hr) [51, 55, 56].

#### 3.2.7. Dexmedetomidine

Dexmedetomidine, a centrally acting *α*_2_-adrenergic agonist, is a recently FDA approved agent used for short term (<24 hours) continuous IV sedation of adults who are tracheally intubated. Like propofol, it has a rapid onset and a relatively rapid elimination half life and is administered as a loading dose followed by continuous IV infusion. One of the advantages is that it provides sedation with a lower risk of respiratory depression than many other sedative medications [57]. There is increased interest with this agent as a sedative during non-invasive neuroradiologic imaging studies in children who are not intubated. In one study, dexmedetomidine was compared to propofol in children undergoing MRI studies. While the onset of sedation and recovery time were significantly shorter in the children that received propofol, hypotension, respiratory depression and desaturation were more common compared to the children receiving dexmedetomidine [58].

There is increased interest in the use of dexmedetomidine as a sedative and potential neuroprotective agent in both adults and children, as animal studies revealed neuroprotection from hypoxia-ischemia and decreased apoptosis and adult human studies in healthy volunteers demonstrated parallel decrease in CMRO_2_ and CBF, which may temporarily be helpful in briefly sedating patients who are at risk for intracranial hypertension such as head trauma, brain tumor, and obstructive hydrocephalus [59, 60]. In pediatric traumatic brain injury case reports, no detrimental effects on their ICP was observed. However, systemic hypertension was observed in one child who were receiving dexmedetomidine with other sedatives, while bradycardia was observed in 2 other children who was receiving dexmedetomidine, other sedatives, and therapeutic hypothermia [59, 60]. Further studies are warranted on the potential use and side effects of this agent in children at risk for intracranial hypertension. 

The most common adverse side effects of dexmedetomidine appear to be cardiovascular. Bradycardia with rare reports of sinus pause or cardiac arrest has been reported. Hypotension has been reported as well as hypertension, the latter thought to be due to peripheral *α*_2B_ agonism with peripheral vasoconstriction. There are conflicting reports on the effects of ventilatory function, with some studies suggesting mild respiratory depression, while others show no effect. While ICP does not appear to increase, cerebral perfusion pressure and CBF have been shown to decrease. The effects on seizure threshold appear to be mixed [61]. Further studies are warranted on the use of this agent in pediatrics.

## 4. Conclusions

While a variety of sedative and analgesic medications have been used in pediatric patients with developmental disabilities and neurologic disorders, it is clear that further studies are needed to determine the optimal agent(s) that will maximize good outcome and will minimize or prevent respiratory, circulatory, and further neurologic compromise.

## Figures and Tables

**Table 1:** 

Agent	Type	Route	Dosage	Sedation/Analgesia
			Induction	

Thiopental	Barbiturate	*IV*	3–8 mg/kg	
		*Rectal*	15–25 mg/kg	

Pentobarbital	Barbiturate	*IV*		2–6 mg/kg (150 mg max)
		Oral		2–6 mg/kg

Ketamine	*N*-methyl-*D*-aspartate (NMDA) antagonist	*IV*	2–4 mg/kg	0.5–1 mg/kg titrated to effect
		*IM*		3–7 mg/kg

Propofol	Hypnotic, Amnestic	*IV*	2–4 mg/kg (Lower dose for critically ill)	Initial: 200–300 mcg/kg/min
				Maintenance Infusion: 125–200 mcg/kg/min (Infants/younger children may require higher dosages)

Etomidate	Hypnotic, Carboxylated imidazole derivative	*IV*	0.3–0.5 mg/kg	

Dexmedetomidine	Central Acting *α*-2 Agonist	*IV*		Loading Dose: 0.5–2 mcg/kg
				Maintenance Infusion: 0.2–0.7 mcg/kg/hour

Morphine	Opioid	*IV*		Bolus: Neonate 25–50 mcg/kg
				Infants and Children 15–30 mcg/kg
				Infusion: Neonate 2–10 mcg/kg/hour
				Infants and Children 15–30 mcg/kg/hour
		*Oral*		Infants and Children 0.25–0.5 mg/kg

Fentanyl	Opioid	*IV*	1–3 mcg/kg	Bolus: 0.5–1 mcg/kg
				Infusion: 0.5–3 mcg/kg/hour
		*Oral*		10–15 mcg/kg
		*Intranasal*		1-2 mcg/kg

Remifentanil	Opioid	*IV*		0.1–0.5 mcg/kg/min

Midazolam	Benzodiazepine	*IV*	0.1-0.2 mg/kg	Bolus: 0.1-0.2 mg/kg
				Infusion 0.1–0.3 mg/kg/hour
		*IM*		0.2–0.5 mg/kg
		*Oral*		0.2–1 mg/kg (max 10 mg)

IV: Intravenous, IM: Intramuscular.
